# Elucidation of Mechanisms of Topotecan-Induced Cell Death in Human Breast MCF-7 Cancer Cells by Gene Expression Analysis

**DOI:** 10.3389/fgene.2020.00775

**Published:** 2020-07-17

**Authors:** Birandra K. Sinha, Erik J. Tokar, Pierre R. Bushel

**Affiliations:** ^1^Immunity, Inflammation, and Disease Laboratory, National Institute of Environmental Health Sciences, Durham, NC, United States; ^2^National Toxicology Program, National Institute of Environmental Health Sciences, Durham, NC, United States; ^3^Biostatistics and Computational Biology Branch, National Institute of Environmental Health Sciences, Durham, NC, United States

**Keywords:** microarray, gene expression, topotecan, ROS, MCF-7

## Abstract

Topotecan is a clinically active anticancer agent for the management of various human tumors. While the principal mechanism of tumor cell killing by topotecan is due to its interactions with topoisomerase I and formation of DNA double-strand breaks, recent studies suggest that mechanisms involving generation of reactive free radicals and induction of oxidative stress may play a significant role in topotecan-dependent tumor cell death. We have shown that topotecan generates a topotecan radical following one-electron oxidation by a peroxidase-hydrogen peroxide system which reacts with reduced glutathione and cysteine, forming the glutathiyl and cysteinyl radicals, respectively. While little is known how these events are involved in topotecan-induced tumor cell death, we have now examined the effects of topotecan short (1 h) and long (24 h) exposure on global gene expression patterns using gene expression microarray analysis in human breast MCF-7 cancer cells, a wild-type p53 containing cell line. We show here that topotecan treatment significantly down-regulated estrogen receptor alpha (ERα/ESR1) and antiapoptotic BCL2 genes in addition to many other p53-regulated genes. Furthermore, 8-oxoguanine DNA glycosylase (OGG1), ferredoxin reductase (FDXR), methionine sulfoxide reductase (MSR), glutathione peroxidases (GPx), and glutathione reductase (GSR) genes were also differentially expressed by topotecan treatment. The differential expression of these genes was observed in a wild-type p53-containing breast ZR-75-1 tumor cell line following topotecan treatment. The involvement of reactive oxygen free radical sensor genes, the oxidative DNA damage (OGG1) repair gene and induction of pro-apoptotic genes suggest that reactive free radical species play a role in topotecan-induced tumor cell death.

## Introduction

**FIGURE 1:**
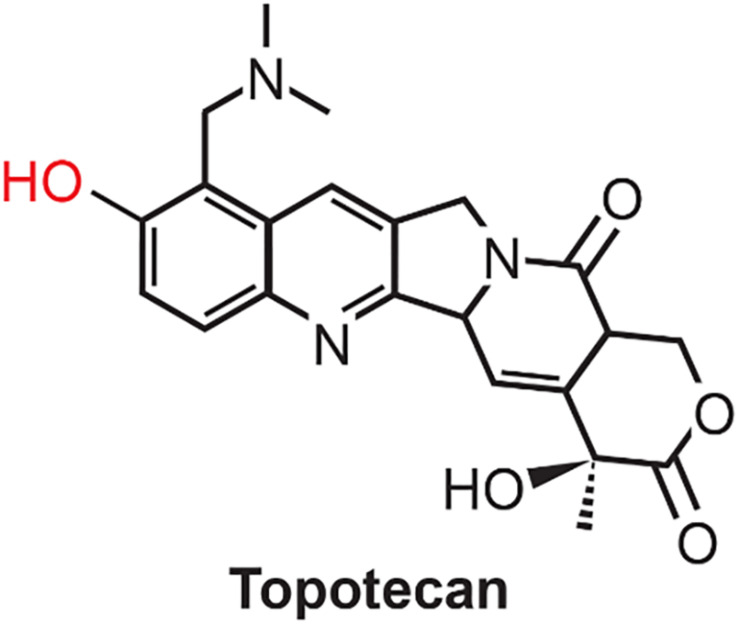
Structure of Topotecan. Phenolic OH, shown in red, is oxidized by peroxidases to generate the corresponding phenoxy radical.

Gene expression profiling is an important tool to understand pharmacological effects of drugs in complex biological systems (Kannan et al., 2001; Daoud et al., 2003; L’Esperance et al., 2008; Januchowski et al., 2017; Qin et al., 2017; Monks et al., 2018). It is now well known that overexpression of certain genes (e.g., wild-type (wt) p53 gene) controls DNA damage, its repair, and cell survival (Levine, 1997; Kannan et al., 2001; Daoud et al., 2003; Januchowski et al., 2017). Topotecan (TPT, Figure 1), a water-soluble analog of camptothecin (CPT), is an important anticancer agent for the treatment of various human malignancies in the clinic (Rowinsky and Verweij, 1997). It is currently used in refractory ovarian cancer as well as for the management of small cell lung carcinoma (Kollmannsberger et al., 1999; Herzog, 2002; Horita et al., 2015). While TPT is not currently utilized for the treatment of breast cancer, it has shown significant activities against breast tumors and is currently used for the treatment of metastatic breast cancer (Oberhoff et al., 2001; Wolff et al., 2005). The major mechanism of action of TPT is believed to results from the formation of highly cytotoxic double-stranded DNA damage produced by the ternary complex formed by DNA-topotecan, and topoisomerase I (Pommier et al., 1994; Sinha, 1995). However, other mechanisms of action of TPT have also been reported e.g., induction of oxidative stress (Akbas et al., 2005; Timur et al., 2005) and inhibition of hypoxia-inducible factor (Rapisarda et al., 2004; Puppo et al., 2008). Various investigators (Akbas et al., 2005; Timur et al., 2005) have reported that TPT induced formation of reactive oxygen species (ROS) in human breast MCF-7 tumor cells. Furthermore, significant decrease in glutathione levels as well as enhancement of lipid peroxidation was observed following TPT treatment. These authors have also found significant increases in levels of antioxidant enzymes, superoxide dismutase, glutathione peroxidase and catalase, following TPT treatment, indicating oxidative stress was induced in MCF-7 tumor cells by TPT. We have recently shown that in the presence of H_2_O_2_ and peroxidases, TPT generates a TPT radical (TPT^•^), which rapidly reacts with glutathione and cysteine, forming the corresponding GS^•^ and Cys^•^ radicals and regenerating TPT (Sinha et al., 2017). It has been shown by several investigators that ROS, produced by arsenic trioxide, and H_2_O_2_ cytotoxicity is partly mediated by the formation of DNA-topo I complexes (Daroui et al., 2004; Sordet et al., 2004). These observations, taken together, suggest that TPT-dependent ROS formation could also contribute to topo I-induced DNA damage and cytotoxicity.

Since a direct effect of free radicals generated by TPT in its cytotoxicity in MCF-7 tumor cells was not addressed in our previous study although we found that that ascorbic acid, a cellular generator of hydrogen peroxide, was extremely synergistic with TPT in inducing cell death (Sinha et al., 2017). To further define the involvement of free radical-mediated pathways in the mechanism (s) in TPT-induced tumor cell death, we have now utilized global gene expression induced by TPT following 1 and 24 h treatment in MCF-7 breast tumor cells. We choose these time points as our preliminary experiments indicated that MCF-7 cells were not affected (showed no sign of cell death) at 1 h and but showed about 35–40% cell death at 24 h of TPT exposure. We believe that examination of differential gene expression at these time points would be indicative of genes and, therefore, pathways and mechanisms responsible for TPT-induced cell death in MCF-7 cells. We found that TPT significantly modulates ERα/ESR1 and BCL2 genes, in addition to several p53-dependent DNA damage repair genes at 24 h of TPT exposure. Furthermore, various ROS sensor genes were also differentially regulated at 24 h by TPT. We also utilized another human breast ZR-75-1 tumor cell line to confirm differential regulations of these genes using RT-PCR. Thus, up-regulation of p53-dependent pathways and down-regulation of ERα/ESR1, BCL2 and ROS sensor genes are extremely important in the mechanism of cytotoxicity of TPT.

## Materials and Methods

### Materials

Topotecan hydrochloride was purchased from Cayman Chemicals (Ann Arbor, MI, United States). TPT was dissolved in doubly distilled water, and solutions were stored at −80°C.

### Cell Culture and Drug Treatment

Human breast MCF-7 (ATCC, Rockville, MD, United States) tumor cells were grown in Phenol Red-free RPMI 1640 media and supplemented with 10% fetal bovine serum and antibiotics. Human breast ZR-75-1 tumor cells (ATCC, Rockville, MD, United States) were grown in Gibco’s DMEM medium containing glucose and glutamine and supplemented with 10% FBS and antibiotics. Both tumor cells were routinely used for 20–25 passages, after which the cells were discarded, and a new cell culture was started from the frozen stock. Three independent experiments of exponentially growing MCF-7 cells (70–75% confluency) were left untreated or treated with TPT (1 μM) for 1 and 24 h. ZR-75-1 cells were treated with TPT (1 μM) for 24 h. Cells were washed twice with PBS (pH 7.4) and total RNA was extracted with TRIzol (Ambion, Life Technologies, Grand Island, NY, United States) and RNeasy mini kit columns (Qiagen, Valencia, CA, United States).

### Global Gene Expression and Data Acquisition

Gene expression analysis was conducted using Agilent Whole Human Genome 4×44K multiplex format oligo arrays (014850) (Agilent Technologies, Santa Clara, CA, United States) following the Agilent 1-color microarray-based gene expression analysis protocol. Starting with 500 ng of total RNA, Cy3 labeled cRNA was produced per manufacturer’s protocol. For each sample, 1.65 μg of Cy3 labeled cRNAs were fragmented and hybridized for 17 h in a rotating hybridization oven. Slides were washed and then scanned with an Agilent Scanner. Data was obtained using the Agilent Feature Extraction software (v12), using the 1-color defaults for all parameters. The Agilent Feature Extraction Software performed error modeling, adjusting for additive and multiplicative noise. The resulting data were processed using OmicSoft Array Studio (Version 10.1) software.

### Preprocessing of the Data

Pixel intensity values of probes that map to the same gene were averaged. The data was log2 transformed and quantile normalized. To remove noisy genes at the low end of the intensity distribution, genes above the 40th percentile in 18 of 24 samples were retained. Thus, 32, 379 genes were filtered down to 18, 248 genes.

### Statistical Data Analysis

The following two-way analysis of variance (ANOVA) was used to model the log2 quantile normalized data from the 18,248 genes passing the low intensity filter:

Y=ijkμ+C+iT+j(C*T)+ijεijk

where μ is grand mean of the experiment, *Y*_*ijk*_ represents the *k*th gene expression observation on the *i*th compound (C) and *j*th time (T) and *ε_*ijk*_* the random error assumed to be normally and independently distributed with mean 0 and standard deviation *δ* for all measurements. Fisher’s least significant difference *t*-test was performed for each gene to compare the mean of the Topo 24 h samples and WT to the mean of the control 24 h samples. Differentially expressed genes (DEGs) were detected at a Benjamini and Hochberg (1995) false discovery rate (FDR)<0.05 and absolute fold change >2.0. Using the Database for Annotation, Visualization, and Integrated Discovery (DAVID) v6.8 (Huang da et al., 2009a, b) the 2,941 DEGs were enriched for KEGG pathways and Gene Ontology biological processes at an FDR ≤0.05 with minimum category size ≥5.

### Pattern-Driven Analysis of Gene Expression

To identify patterns in 18,248 filtered genes, ratio values for each gene were generated by subtracting the average log2 quantile normalized pixel intensity of the time-matched controls from the log2 quantile normalized pixel intensity of the time-matched samples. The ratio values were then analyzed to extract patterns and identify co-expressed genes using the EPIG software (Chou et al., 2007) with the EPIG parameters Pearson correlation ≥0.7, signal/noise ≤2.5, minimum pattern size ≥6, magnitude of fold change >0.5 and *p*-value < 0.0001.

### Real-Time RT-PCR

The expression levels of selected transcripts were confirmed by real-time polymerase chain reaction (RT-PCR) using absolute SYBR green ROX Mix (Thermo Fisher Scientific, Rochester, NY, United States). Data were analyzed using ΔΔCt method of relative quantification in which cycle times were normalized to β-actin from the same sample. Primers for the selected genes were designed using Primer Express 1.0 software and in some cases were synthesized (Integrated DNA Technologies, San Diego, CA, United States) from published literature. All real-time fluorescence detection was carried out on an iCycler (Bio-Rad, Hercules, CA, United States). Statistical analysis represents the mean ± SEM from three independent experiments and were performed using unpaired Student’s *t*-test and considered significant when *p* ≤ 0.05.

## Results

Enrichment analysis of the 2,197 genes (2,604 transcripts, Supplementary Table 1) showing significant differences between 24 h-treated MCF-7 tumor cells and vehicle-treated controls are presented in Tables 1–3. Using KEGG pathways, the gene ontology (GO) biological process and Ingenuity Pathway analysis (IPA), p53 signaling pathway, DNA replication, and positive regulator of apoptotic process were identified to be significantly enriched. We also found that DEGs involved in DNA repair pathways were also over-expressed following TPT treatment.

**TABLE 1:** Enrichment of biological pathways by the TPT at 24 h differentially expressed genes.

KEGG pathway	KEGG Term	Gene Count	Pop Hits	%	Fold Enrichment	FDR (%)
p53 signaling pathway	hsa04115	29	67	1.37	3.79	2.4E-07
Cell cycle	hsa04110	39	124	1.84	2.76	6.3E-06
DNA replication	hsa03030	19	36	0.90	4.62	1.4E-05

*Based on KEGG enrichment of the 2,197 DEGs (2,604 transcripts) using the David Database v6.8. The population (pop) total = 6,879 and the list total = 785. FDR: false discovery rate.*

**TABLE 2:** Enrichment of biological processes by the TPT at 24 h differentially expressed genes.

BP	BP Term	Gene Count	Pop Hits	%	Fold Enrichment	FDR (%)
DNA replication	GO:0006260	53	155	2.50	3.18	3.38E-11
G1/S transition of mitotic cell cycle	GO:0000082	35	102	1.65	3.19	1.37E-06
Cellular response to DNA damage stimulus	GO:0006974	51	208	2.41	2.28	5.70E-05
Cell division	GO:0051301	71	350	3.35	1.89	3.50E-04
DNA replication initiation	GO:0006270	16	32	0.75	4.65	4.99E-04
DNA repair	GO:0006281	53	235	2.50	2.10	5.31E-04
Positive regulation of GTPase activity	GO:0043547	100	565	4.72	1.65	0.001
Telomere maintenance via recombination	GO:0000722	15	32	0.71	4.36	0.004
Positive regulation of apoptotic process	GO:0043065	60	300	2.83	1.86	0.006
DNA damage response, signal transduction by p53 class mediator resulting in cell cycle arrest	GO:0006977	21	62	0.99	3.15	0.008
CENP-A containing nucleosome assembly	GO:0034080	17	43	0.80	3.68	0.009
G2/M transition of mitotic cell cycle	GO:0000086	34	137	1.60	2.31	0.012
Sister chromatid cohesion	GO:0007062	28	103	1.32	2.53	0.015
Signal transduction	GO:0007165	171	1161	8.07	1.37	0.023
Mitotic nuclear division	GO:0007067	50	248	2.36	1.88	0.033

*Based on GO biological process (BP) enrichment of the 2,197 DEGs (2,604 transcripts) using the David Database v6.8. The population (pop) total = 16,792 and the list total = 1,804. FDR: false discovery rate.*

**TABLE 3:** Canonical pathways impacted by the TPT at 24 h differentially expressed genes.

Ingenuity canonical pathways	Ratio	z-score	−log(*p*-value)
p53 signaling	0.28	2.71	7.89
Molecular mechanisms of cancer	0.18	NaN	7.15
Estrogen-mediated S-phase Entry	0.46	−2.31	5.98
Cell cycle: G2/M DNA damage checkpoint regulation	0.34	1	5.91
Role of BRCA1 in DNA damage response	0.28	−0.54	5.67
Mitotic roles of polo-like kinase	0.29	0.26	5.3
Cell cycle control of chromosomal replication	0.30	NaN	5.15
Breast cancer regulation by stathmin1	0.19	NaN	4.68
NER pathway	0.22	−0.23	4.28
Axonal guidance signaling	0.15	NaN	4.24
Cell cycle: G1/S checkpoint regulation	0.25	1.07	4.04

*NaN, No activity pattern detected.*

Table 4 shows a list of some of the DNA repair genes significantly affected following TPT treatment (24 h). Of interest are significant decreases (4.5-fold) related to DNA damage repair genes, e.g., O^6^-methylguanine-DNA methyl transferase (*MGMT*) gene and 8-oxoguanine DNA glycosylase (*OGG1*) gene following TPT treatment. *MGMT* is responsible for the removal (and repair) of alkyl groups from DNA and protects cells from cytotoxic effects of alkylating anticancer drugs. *OGG1* is involved in the repair of 8-oxoguanine, formed from reactions of hydroxyl radical with DNA. DNA repair gene, p53-dependent *RAD51* was also significantly decreased (4.0-fold) following TPT treatment. *RAD51* is known to be involved in homologous repair of DNA double strand breaks following DNA damage in a p53-dependent mechanism (Arias-Lopez et al., 2006; Hannay et al., 2007; Nogueira et al., 2011). The growth arrest and DNA damage 45 alpha gene (*GADD45α*) was significantly (16.8-fold) induced. GADD45α is a stress and DNA damage response protein and is increased during growth arrest following treatment with DNA damaging drugs.

**TABLE 4:** DNA repair genes differentially regulated by TPT 24 h treatment in MCF-7 tumor cells.

GenBank Acc. #	Probe ID	Gene Symbol	Description	Log2 Fold-Change
NM_016819, NM_002542	A_24_P414183, A_23_P344392	OGG1	8-oxoguanine DNA glycosylase	−2.1
NM_006763	A_23_P62901	BTG2	BTG family, member 2	5.0
NM_000057	A_23_P88630	BLM	Bloom syndrome, RecQ helicase-like	−2.6
NM_000123	A_23_P117225	ERCC5	excision repair cross-complementing rodent repair deficiency, complementation group 5	2.3
NM_005244	A_23_P500421	EYA2	eyes absent homolog 2 (Drosophila)	−5.8
NM_000135	A_23_P206441	FANCA	Fanconi anemia, complementation group A	−2.7
NM_000136	A_23_P32021	FANCC	Fanconi anemia, complementation group C	−6.4
NM_001018115	A_32_P24165	FANCD2	Fanconi anemia, complementation group D2	−3.6
NM_018193	A_32_P95729	FANCI	Fanconi anemia, complementation group I	−3.1
NM_002431	A_23_P32615	MNAT1	menage a trois homolog 1, cyclin H assembly factor (Xenopus laevis)	−2.3
NM_145080	A_23_P95823	NSMCE1	non-SMC element 1 homolog (S. cerevisiae)	−2.1
NM_002412	A_23_P104323	MGMT	O-6-methylguanine-DNA methyltransferase	−4.5
NM_148894	A_24_P264928	BOD1L	biorientation of chromosomes in cell division 1-like	3.8
NM_001274	A_23_P116123	CHEK1	checkpoint kinase 1	−2.3
NM_005483	A_24_P53519	CHAF1A	chromatin assembly factor 1, subunit A (p150)	−2.9
NM_005441	A_23_P57306	CHAF1B	chromatin assembly factor 1, subunit B (p60)	−2.2
NM_000107	A_23_P52610	DDB2	damage-specific DNA binding protein 2, 48kDa	2.0
NM_003686	A_23_P23303	EXO1	exonuclease 1	−3.1
NM_001136198	A_23_P142325	FZR1	fizzy/cell division cycle 20 related 1 (Drosophila)	2.3
NM_004111	A_24_P84898	FEN1	flap structure-specific endonuclease 1	−2.2
NM_202002	A_23_P151150	FOXM1	forkhead box M1	−5.2
NM_001924	A_23_P23221	GADD4A	growth arrest and DNA-damage-inducible, alpha	16.8
NM_002439	A_24_P203479	MSH3	mutS homolog 3 (E. coli)	−2.1
NM_001031716	A_24_P229531	OBFC2A	oligonucleotide/oligosaccharide-binding fold containing 2A	2.5
NM_198949	A_23_P134295	NUDT1	nudix (nucleoside diphosphate linked moiety X)-type motif 1	−2.1

**FIGURE 2:**
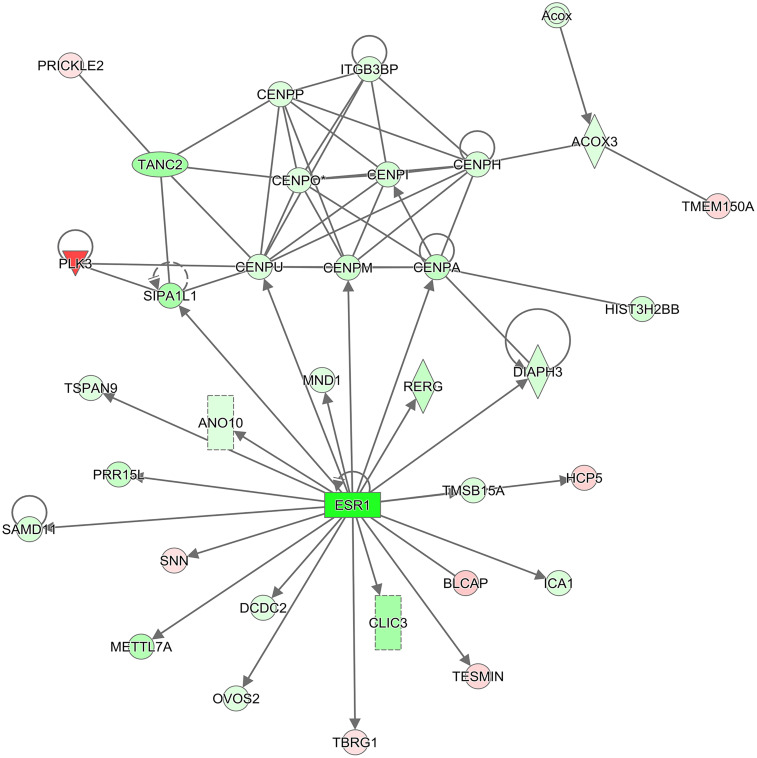
IPA molecular interaction network. The 2,197 DEGs (2,604 transcripts) from the comparison of Topotecan at 24 h vs. control at 24 h were used to generate the top scoring (–log_10_ *p*-value = 38) molecular interaction network containing 34 focus molecules. The intensity of the colors represents the fold change of expression; red is up-regulated, and green is down-regulated. The shapes represent molecular entities; diamond: enzyme, oval: transcription regulator, triangle: kinase, rectangle: ligand-dependent nuclear receptor, double circle: complex group, circle: other. Solid lines denote a direct interaction. An arrow symbolizes activation and a line with no arrow signifies a protein-protein interaction.

BCL2, the anti-apoptotic protein, was significantly decreased (8.2-fold) following TPT treatment in the breast MCF-7 tumor cells, suggesting an apoptosis-based cell death. Furthermore, a significant decrease in the ERα receptor gene was also detected following TPT treatment. Interestingly, IPA enrichment of DEGs at 24 h (Table 4) shows a central role for ERα receptor and its interactions with various genes involved in TPT-induced cell death (Figure 2). This conclusion is also supported by a previous study (Punchihewa et al., 2007) involving treatment of MCF-7 cells with a topoisomerase inhibitor (XR5944), like topotecan, where authors found significant inhibition of ESR1 activity. In our Ingenuity molecular interaction network (Figure 2), many of the DEGs from topotecan exposure at 24 h interact with ESR1 as a central hub. ESR1 is down-regulated as are the majority of the genes in the network.

**FIGURE 3:**
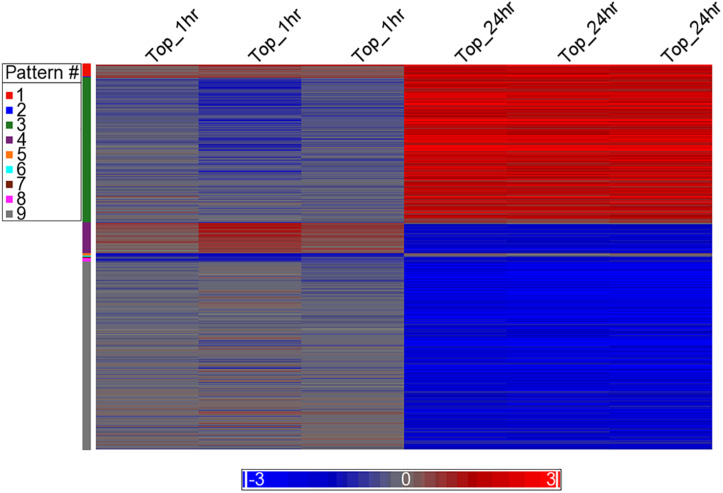
Heat map of the expression of the co-expressed genes. The log_2_ ratio values of the 1,132 co-expressed genes categorized to the nine EPIG patterns are represented as a heat map. Red is up-regulated, blue is down-regulated, and gray is no change. The color legend depicts the relative expression change (each sample to the average of the time-matched control). The *x*-axis is the samples and the *y*-axis are the genes grouped into the nine patterns colored according to the pattern # legend.

**FIGURE 4:**
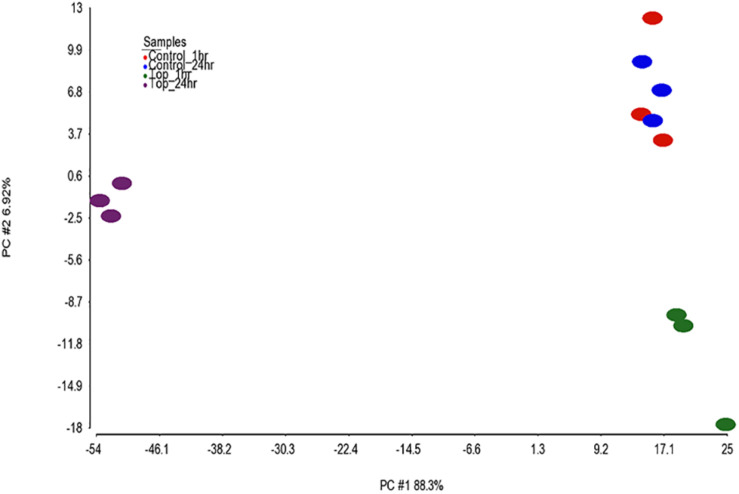
PCA of the samples. PCA was performed on the 1,132 EPIG co-expressed genes using log2 ratio values (expression of each topotecan sample or control minus the average of the log2 of the time matched controls). The total amount of variability captured by the 1st two principal components (PCs) is 95.2%. The *x*-axis is PC1 (88.3%) and the *y*-axis is PC2 (6.92%). The samples are color coded as depicted in the legend.

**FIGURE 5:**
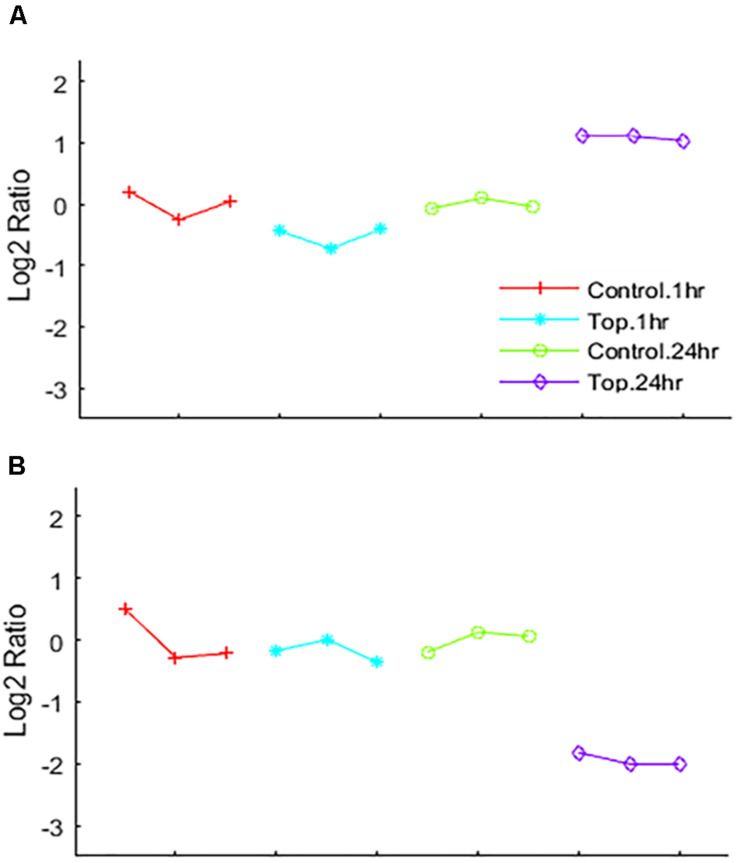
EPIG patterns. Expression patterns of genes co-expressed by **(A)** up-regulation by Topotecan (pattern #3 represents 426 genes) and **(B)** down-regulation by Topotecan (pattern #9 represents 552 genes). The *x*-axis is the biological samples that are color coded and represented by symbols as depicted in the legend. The *y*-axis is the log_2_ ratio.

Differential analysis of the 1 h treated samples yielded no significant changes in genes. However, 1,132 gene transcripts were identified as co-expressed in nine patterns across the 1 and 24 h treatments (Figure 3 and Supplementary Table 2). These co-expressed genes by principal component analysis (PCA) capture more than 96% of the variation in the data, the co-expressed gene group the biological replicates very well and separate the vehicle treated controls (1 and 24 h) from the TPT-treated samples (Figure 4). Furthermore, the 1st principal component (PC #1) separates 24 h TPT-treated samples from the controls and the 1 h TPT-treated samples. Most co-expressed genes are in pattern #3 (426 transcripts) and pattern #9 (552 transcripts). As shown in Figures 3, 5, pattern #3 genes are up-regulated at the 24 h TPT treatment and the pattern #9 genes down-regulated at the 24 h TPT treatment. Pattern #3 genes enrich for the p53 signaling pathway and pattern # 9 genes enrich for DNA replication and cell cycle pathways.

**FIGURE 6:**
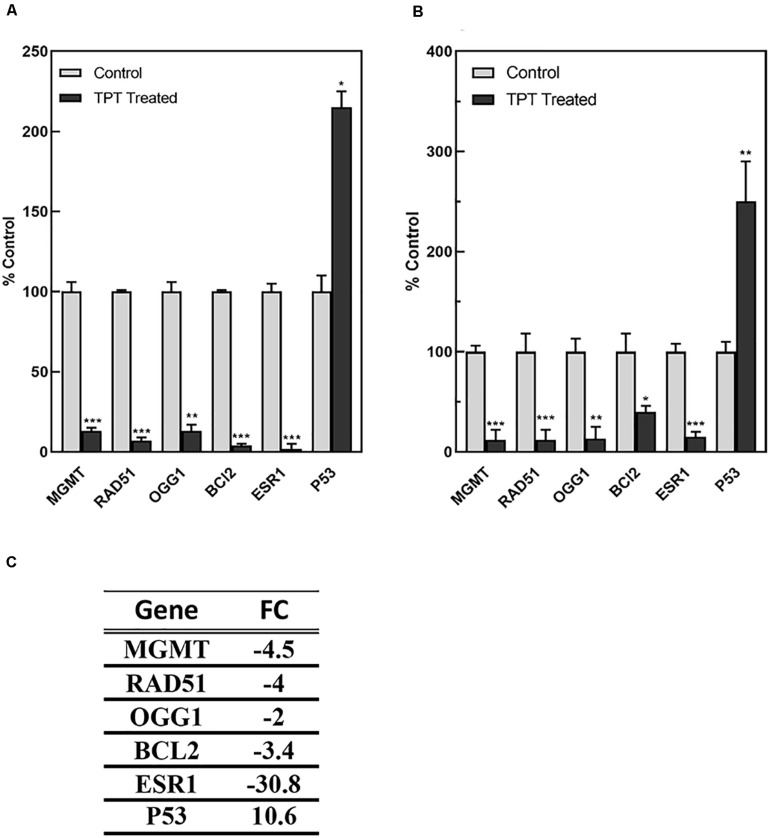
RT-PCR analysis of representative genes regulated by TPT in MCF-7 **(A)** and ZR-75-1 **(B)** breast tumor cells at 24 h.***, **, and * *p* values <0.0001, <0.005, and 0.05, respectively, compared to controls. The data in **(C)** was obtained by microarray analysis for MCF-7 cell line and is expressed as the fold change from controls.

As TPT treatment at 1 h did not significantly affected expressions of genes, we used the RT-PCR analysis to confirm expression of these representative (from microarray analysis) genes at 24 h in MCF-7 tumor cells (Figure 6). A significant correlation was found between microarray (C) and RT-PCR data (A) for MCF-7 cells. Our RT-PCR studies in ZR-75-1 cells, a wild-type p53-containg human breast tumor cell line, indicated similar results, confirming results obtained in MCF-7 tumor cells (Figures 6A,B). Our results strongly suggest that these genes are similarly regulated by TPT in both cell lines.

The microarray analysis also indicated that various oxy-radical sensor genes were also significantly differentially expressed by TPT treatment of MCF-7 breast cancer cells at 24 h. We used RT-PCR then to confirm differential expressions of oxy-radical sensor genes in MCF-7 breast tumor cells. Again, RT-PCR was utilized to examine the effects of TPT on these various oxy-radical sensor genes in ZR-71-1 tumor cells and data in Table 5 clearly show that there is a significant correlation with microarray and RT-PCR in both MCF-7 and ZR-75-1 cells. Ferredoxin reductase (FDXR) is reported to be involved in p53-mediated apoptosis via generation of ROS in mitochondria (Hwang et al., 2001). Glutathione peroxidases (GPx) are selenium containing cellular proteins responsible for detoxifications of hydrogen peroxide and lipid peroxides. Data presented in Table 5 clearly show a significant correlation with data obtained with microarray analysis and RT-PCR data obtained with both MCF-7 and ZR-75-1 tumor cells.

**TABLE 5:** Oxy-radical sensor genes differentially up-regulated/down-regulated following TPT exposure (24 h) in MCF-7 and ZR-75-1 breast tumor cells.

Gene Symbol	FC^a^	FC^b^	FC^c^
FDXR	6.4	3.5	5.0
MSRA	−5.8	−5.0	−6.0
GSR	−2.2	−2.1	−2.0
GPx	2.0	4.0	5.0

*^a^microarray in MCF-7 cell line; ^b^RT-PCR in MCF-7 cell line; ^c^RT-PCR in ZR-75-1 cells line.*

## Discussion

The main mechanism of TPT-dependent tumor cell killing is based on its known interactions with topoisomerase I that results in the stabilization of DNA-enzyme-drug complexes and the formation of toxic DNA double-strand breaks (Pommier et al., 1994; Sinha, 1995). While this remains one of the most accepted mechanism of tumor cell death by TPT, several studies indicate that generation of ROS and oxidative stress may also be important in TPT-induced cell death (Akbas et al., 2005; Timur et al., 2005).

Recently, we used MCF-7 breast cancer cells to show that TPT is readily oxidized to a free radical intermediate (TPT^•^) and we found that ascorbic acid, a cellular generator of hydrogen peroxide, was extremely synergistic with TPT in inducing cell death (Sinha et al., 2017). One of the main reasons for utilizing MCF-7 tumor cells to identify free radical based pathways for TPT-induced tumor cell death was that we have shown that MCF-7 breast tumor cells are extremely efficient in activating adriamycin to its free radical species and these cells contain significant numbers of free radical-based detoxification enzymes e.g., SOD, catalase and GPx (Sinha et al., 1987, 1989). In this study, we used microarray gene expression analysis to identify and delineate the roles of various genes responsible for TPT cytotoxicity in human MCF-7 breast tumor cells. We have identified several genes belonging to the p53 signaling pathway, DNA replication, and positive regulators of apoptotic process that were differentially expressed by TPT treatment. Using microarray differential gene expression analysis, Davis et al. (2015) have reported that topotecan exposure in rat bone marrow resulted in enrichment of cell cycle and DNA replication pathways similar to our observations in this study. It should be noted that the relationship between chromatin remodeling, cell cycle and DNA repair factors are usually not discussed in relation to DNA damage response mechanisms following topotecan exposure. Our analysis described here clearly shows a possible relationship between chromatin remodeling complexes and mechanisms of topotecan toxicity and repair that needs further investigation.

Microarray analysis indicated that several genes involved in DNA damage repair pathways were differentially expressed. We, therefore, utilized RT RT-PCR analysis to confirm results obtained by the microarray analysis in MCF-7 tumor cells. Furthermore, these findings were confirmed in another human wt53-conaining ZR-75-1 breast tumor cell line by RT-PCR. We found that TPT treatment caused significant decreases in both O^6^-methylguanine-DNA methyl transferase (*MGMT*) and 8-oxoguanine DNA glycosylase (*OGG1*) genes. *MGMT* is responsible for the repair of alkyl groups from O^6^-guanine and protects cells from cytotoxic effects of alkylating anticancer drugs, e.g., BCNU and temozolomide (TMZ) (Gerson, 2004; Hegi et al., 2005; Zhang et al., 2012; Thomas et al., 2017). TMZ is currently used for the treatment of glioblastoma multiforme. TMZ is rapidly converted to an alkylating species and forms O^6^-methylguanine for its cytotoxicity. Therefore, the formation and persistence of O^6^-methylguanine is critical for its toxicity. It has been reported that TMZ is significantly more cytotoxic to cells with low MGMT activity and increases in MGMT expression have been shown to play a key role in the development of resistance to TMZ and other similar O^6^-alkylating drugs (Ostrowski et al., 1991; Nagane et al., 1992). Because of the potential of TMZ for the treatment of glioblastoma and other malignancies e.g., metastatic melanoma, it would be ideal to combine TMZ with other anticancer agents. Since our present study demonstrates that treatment of tumors with TPT significantly decreases the expression of *MGMT*, it would be of interest to combine TMZ with TPT for synergistic tumor cell killing. Preliminary studies indicate that co-treatment of MCF-7 tumor cells with TPT (125 nM) with TMZ (100 μM) for 48 h results in synergistic tumor cells death. Under these conditions we found 35–40% cell death with this combination of TPT and TMZ while very little or no cells death was observed with either agent alone. MCF-7 tumor cells are extremely resistant to TMZ due to high MGMT activities. Further studies are currently under active investigation in our laboratory using various mer+ tumors cells, including human brain tumor cell lines which overexpress *MGMT* (Ostrowski et al., 1991; Nagane et al., 1992).

8-Oxoguanine glycosylase (OGG1) is involved in the repair of 8-oxoguanine in DNA which is formed from the oxidation of DNA with hydroxyl radical (OH), generated from the H_2_O_2_ by metal ion catalysis (Grollman and Moriya, 1993; Valavanidis et al., 2009). Formation of 8-oxoguanine in cells has been implicated in oxidative stress. In the present study, *OGG1* was significantly decreased by TPT treatment, suggesting decreased repair of OH radical-mediated 8-oxoguanine and enhanced tumor cell killing. Increases in OGG1 activity have been reported to result in the development/emergence of resistance to ionizing radiation. It is interesting to note that TPT and ionizing radiation act synergistically in inducing tumor cell death (Mattern et al., 1991; Lamond et al., 1996; Chen et al., 1999). Various anticancer drugs, including doxorubicin and arsenic-containing drugs generate ROS and form 8-oxyguanine in tumor cells (Piedade et al., 2002; Pu et al., 2007). It would, therefore, be ideal to combine TPT with other free radical generating anticancer drugs for enhanced/synergistic tumor cell killing. It is possible that enhanced MCF-7 tumor cell killing observed with TPT and ascorbic acid observed in our previous study may, in part, have resulted from this decrease in OGG1 by TPT (Sinha et al., 2017).

The present study indicated a significant upregulation of ferredoxin reductase (*FDXR*) and a significant down-regulation of methionine sulfoxide reductase (*MSR*) genes following TPT treatment in both MCF-7 and ZR-75-1 tumor cells. The role of *FXDR* in free radical biology is well established as it has been reported to be involved in p53-mediated apoptosis via generation of ROS in mitochondria (Hwang et al., 2001), causing oxidative stress in cells. Normal and tumor cells have various protective mechanisms, including induction of cellular antioxidant enzymes (e.g., SOD, catalase, and various peroxidases) as well as DNA repair enzymes for oxidative DNA damage (e.g., glycosylases). Both free and bound methionine are readily oxidized by ROS. In cellular systems, methionine is oxidized to methionine sulfoxide, altering protein confirmation and/or inactivating proteins (Romsang et al., 2013). Methionine sulfoxide is reduced by methionine sulfoxide reductase (MSR) to methionine using NADPH/thioredoxin reductase/thioredoxin system (Romsang et al., 2013). Decreases or inactivation of MSR increases sensitivity to ROS-mediated cell killing and has been suggested to be involved in the killing of neutrophils by HOCL, a powerful oxidant inducing oxidation of methionine (Moskovitz et al., 1996; Boschi-Muller et al., 2008; Rosen et al., 2009). We also found a small but a significant decrease in the glutathione reductase gene (GSR) following TPT exposure in MCF-7 tumor cells. GSR is required for the reduction of oxidized glutathione (GSSG) that is formed from the reaction of GSH with increased ROS in cells. Furthermore, TPT treatment caused increases in glutathione peroxidase (GPx) which are selenium containing enzymes that are involved in the removal of peroxides, including lipid peroxides from cells. Modulation of both *GPx* and *GSR* genes have been implicated in the oxidative stress induced by CCl4 in hepatocytes (Iskusnykh et al., 2013).

Taken together e.g., modulations of *FXDR*, *MSR*, *GSR*, and *GPx* genes by TPT in MCF-7 (and ZR-75-1) tumor cells strongly indicate formation of ROS and induction of cellular imbalance of homeostasis, resulting in increased oxidative stress by TPT. This would lead to the formation of cytotoxic species and damage to critical cellular macromolecules. This is further supported by our results showing a significant decrease in *OGG1* gene expression, the DNA repair enzyme for 8-oxoguanine, indicating decrease in repair of oxidant-dependent damaged DNA. These results indicate that increased cellular ROS formation and resulting oxidative stress may have implications in the TPT-induced tumor cell death.

**FIGURE 7:**
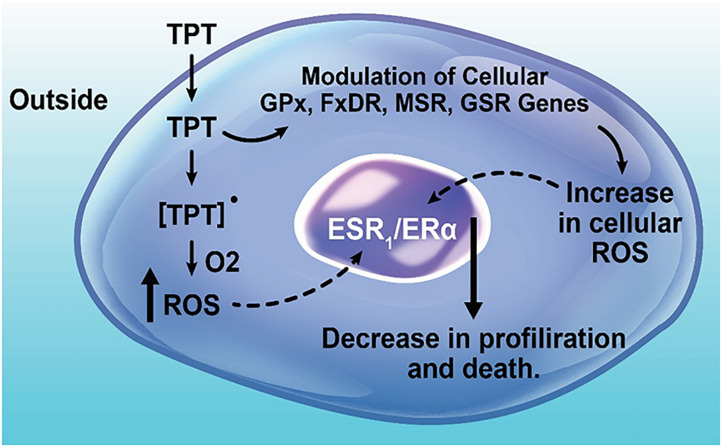
Topotecan-induced modulation of free radical sensing genes, up-regulation of ROS production, and regulation of cell proliferation and death.

Finally, our studies indicate a significant down-regulation of ERα/ESR1 by TPT treatment. It is interesting to note that XR5944, a topoisomerase I poison, has also been reported to down-regulate *ESR1*, similar to our observations. A significant amount of published data would suggest that ERα/ESR1 is involved in apoptosis, cell death and drug resistance (Mobley and Brueggemeier, 2004; Sui et al., 2007; Bailey et al., 2012; Cook et al., 2014; Zhou and Zhu, 2014). Cook et al. (2014) have shown that the knock-down of ERα receptor leads to ROS-mediated autophagy and cell death in breast cancer cells. In addition, studies of Mobley and Brueggemeier (2004) indicate that treatment of MCF-7 breast cancer cells with estrogen decreases the cell’s ability to metabolize H_2_O_2_, resulting in increased sensitivity to DNA damage by peroxides. It is also interesting to note that this treatment of MCF-7 cancer cells also resulted in decreased levels of GSH, and increased activities of both GPx and SOD (Mobley and Brueggemeier, 2004), results which are similar to those reported in this manuscript with TPT treatment of both MCF-7 and ZR-75-1 cancer cells. Taken together, these observations suggest that ERα is directly involved in free radical mediated tumor cell death. It should also be noted that the antiestrogen drug Tamoxifen is highly synergistic with TPT in cells and in the clinic, indicating that ERα receptor signaling is involved in TPT-induced cell death in ERα positive tumor cells (Sugimoto et al., 2003; Morgan et al., 2010; Khuroo et al., 2014). BCL2, the anti-apoptotic protein was significantly decreased following TPT treatment in the MCF-7 tumor cells, suggesting an apoptosis-based cell death. Based on the heat map (Figure 3) of genes up-regulated and down-regulated following TPT treatment, and the Ingenuity Pathway Analysis of the differentially expressed genes (DEGs) at 24 h, a central role for ERα receptor and its interactions with various genes involved in TPT-induced cell death is apparent (Figure 2). Based on these findings, a possible ROS-dependent mechanism of tumor cell death by TPT is proposed and is shown in Figure 7.

## Conclusion

Our study shows that treatment of human breast MCF-7 tumor cells with TPT results in DEGs that regulate p53-dependent pathways as well as genes that regulate apoptosis. Our results also indicate a central role of ERα in TPT cytotoxicity. Furthermore, expressions of DNA damage repair genes, *MGMT* and *OGG*, were found to be significantly decreased following TPT treatment. Our study has further identified several genes, *FDXR, MSR, GSR*, and *GPx*, involved in maintenance of cellular homeostasis due to increased ROS formation, were differentially expressed by TPT, indicating a putative role of ROS and oxidative stress in TPT cytotoxicity.

## Data Availability Statement

The datasets generated for this study can be found in the NCBI’s Gene Expression Omnibus (GEO) and are accessible through GEO Series accession number GSE138442 (https://www.ncbi.nlm.nih.gov/geo/query/acc.cgi?acc=GSE138442).

## Author Contributions

BS conceived and designed the experiments. BS and ET performed the experiments. BS, ET, and PB analyzed the data. BS, ET, and PB wrote the manuscript. All authors contributed to the article and approved the submitted version.

## Disclaimer

Statements contained herein do not necessarily represent the statements, opinions, or conclusions of NIEHS, NIH, or the US Government.

## Conflict of Interest

The authors declare that the research was conducted in the absence of any commercial or financial relationships that could be construed as a potential conflict of interest.

## Supplementary Material

TABLE S1 Excel spreadsheet containing the TPT 24 h treatment differentially expressed genes.

TABLE S2 Excel spreadsheet containing the TPT 24 h treatment EPIG down-regulation genes categorized to pattern #9.

## Abbreviations

BCL2: B cell lymphoma 2
ER α /ESR1: estrogen receptor alpha
FDXR: ferredoxin reductase
GPx: glutathione peroxidases
GSR: glutathione reductase
MGMT: O^6^-methylguanine-DNA methyltransferase
MSR: methionine sulfoxide reductase
OGG1: 8-oxoguanine DNA glycosylase1
ROS: reactive oxygen species
RT-PCR: real-time polymerase chain reaction
TPT: topotecan.
